# A case of relapsed gAChR-positive autoimmune autonomic ganglionopathy treated by plasma exchange and mycophenolate mofetil

**DOI:** 10.3389/fneur.2024.1533840

**Published:** 2025-01-10

**Authors:** Zhijie Lu, Xiaojie Cao, Mingyu Wang, Fang Peng, Lin Chen, Zegang Yin, Baiyang Zheng, Jin Fan, Mingjie Zhang

**Affiliations:** Department of Neurology, The General Hospital of Western Theater Command, Chengdu, Sichuan, China

**Keywords:** autoimmune autonomic ganglionopathy, ganglionic nicotinic acetylcholine receptor, orthostatic hypotension, gastrointestinal dysfunction, case report

## Abstract

Autoimmune autonomic ganglionopathy (AAG) is a rare and acquired immune-mediated disease that leads to wide autonomic failure, mainly characterized by orthostatic hypotension, gastrointestinal dysfunction, anhidrosis and poorly reactive pupils. This disorder is usually associated with autoantibodies to the ganglionic nicotinic acetylcholine receptor (gAChR-Ab). In this study, we describe a case of a gAChR-Ab-positive AAG patient with two therapeutic stages. The patient responded well after the first stage of methylprednisolone pulse therapy and subsequent low-dose prednisone. However, AAG relapsed after stopping oral prednisone. In the second stage, repeated methylprednisolone pulse therapy was less effective than before. Fortunately, multiple plasma exchange treatments improved the patient’s symptoms. In the end, low-dose oral prednisone and mycophenolate mofetil provided significant improvement in this patient during long-term follow-up. AAG is a relatively rare neuroimmunological disease with insidious onset and confused clinical features, while it responds well to the conventional immunotherapy, and some patients may require a long-term immunotherapy. Emphasizing the importance of early detection and treatment in clinical practice. Moreover, it should be noted that the reduction and withdrawal of immunosuppressants should be slow and cautious.

## Introduction

Autoimmune autonomic ganglionopathy (AAG) is an uncommon immune-mediated disease of the autonomic nervous system, characterized by significant orthostatic hypotension, severe gastrointestinal dysfunction, anhidrosis and poorly reactive pupils (1). Autoantibodies to ganglionic acetylcholine receptors (gAChR-Ab), as the serological markers, are found in about half of the patients with AAG (2). Many patients can benefit from the immunotherapies such as corticosteroid, intravenous immunoglobulin and plasma exchange (3). In this current report, we describe a case of a gAChR-Ab-positive AAG patient with two therapeutic stages. In the initial stage, corticosteroid treatment was effective, but symptoms relapsed after drug withdrawal. Repeated methylprednisolone pulse therapy was less effective than before. Combination treatment of corticosteroid and subsequent plasma exchange had good curative effects. Finally, a low dose of corticosteroids and immunosuppressants provided significant improvement in this patient during long-term follow-up. The present patient differs from many reported Chinese cases with gAChR antibody-negative/lacking, which typically have a unidirectional disease course and prognosis. AAG is a relatively rare neuroimmunological disease with insidious onset and confused clinical features, while it is a treatable disease with a good prognosis. Emphasizing the importance of early detection and treatment in clinical practice.

## Case presentation

### First stage

A 54-year-old Chinese woman was admitted to the Department of Neurology of our hospital on 8 March 2022, with a main complaint of multiple episodes of consciousness disturbance within the past year. One year before admission, the patient suddenly fainted at work without consciousness for 1–2 min. After waking up, she experienced temporary limb numbness and weakness but returned to normal soon, without convulsions, foaming at the mouth, urinary and fecal incontinence. Over the past year, she intermittently experienced similar symptoms, mostly after activity. Multiple tests including electrocardiograph (ECG) and cranial CT showed no abnormalities. The cause of recurrent syncope was still unknown. Three days before admission, episode of syncope was more frequent than before, which occurred 3–5 times a day. Half year ago, she underwent gastroscopy which indicated gastric retention, complaining of gastrointestinal symptoms such as recurrent abdominal distension, vomiting, diarrhea, and constipation. There was no history of infectious diseases or other chronic illnesses; personal, menstrual, and family histories were unremarkable.

**Figure 1:**
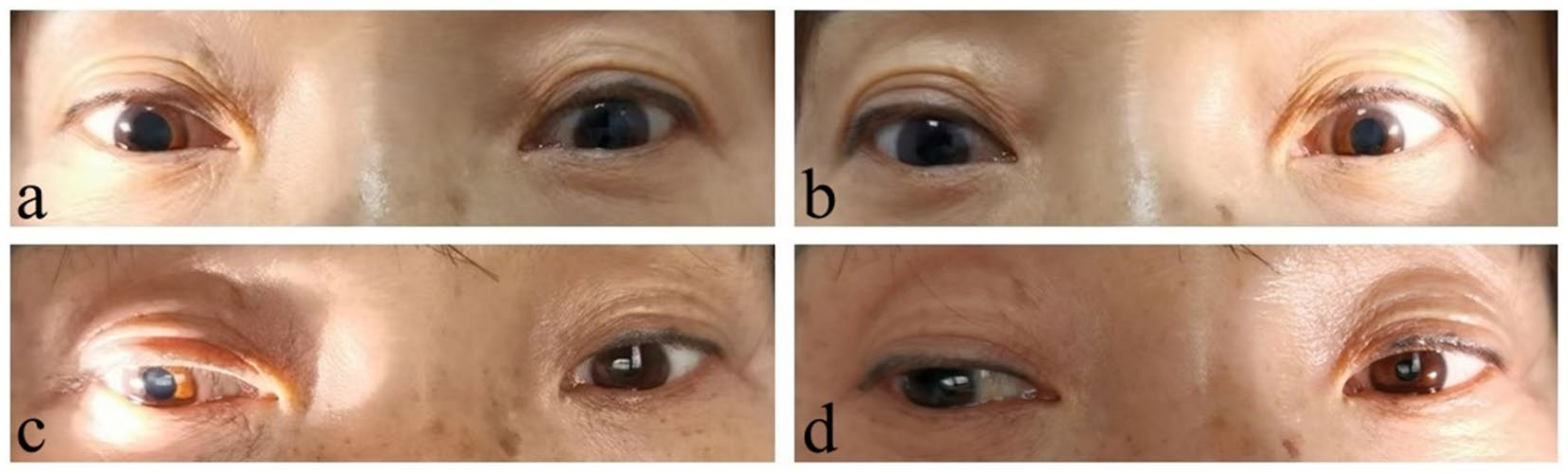
Pupil signs compared before and after treatment. Before treatment, bilateral pupils were dilated with absent responses to light (**A**, right side; **B**, left side). After treatment, the pupils were still slightly dilated, but the pupillary reaction to light shown some improvement, especially the left side (**C**, right side; **D**, left side).

Upon admission, physical examination showed a body temperature of 36.2°C, a heart rate of 60 beats/min, a respiration rate of 18 breaths/min, and a supine blood pressure of 146/90 mmHg, and a standing blood pressure of 64/44 mmHg. Heart and lung examinations were negative. Abdominal examination showed slight distension without significant tenderness or rebound tenderness. Neurological examination revealed clear consciousness and fluent speech. Bilateral pupils were approximate 5.0 mm in diameter with absent responses to light and accommodation, while no significant abnormalities in other cranial nerves (Figures 1A,B). She had normal muscle strength and tone. There were no sensory deficits and abnormal tendon reflexes. The bilateral Babinski signs were negative.

**Figure 2:**
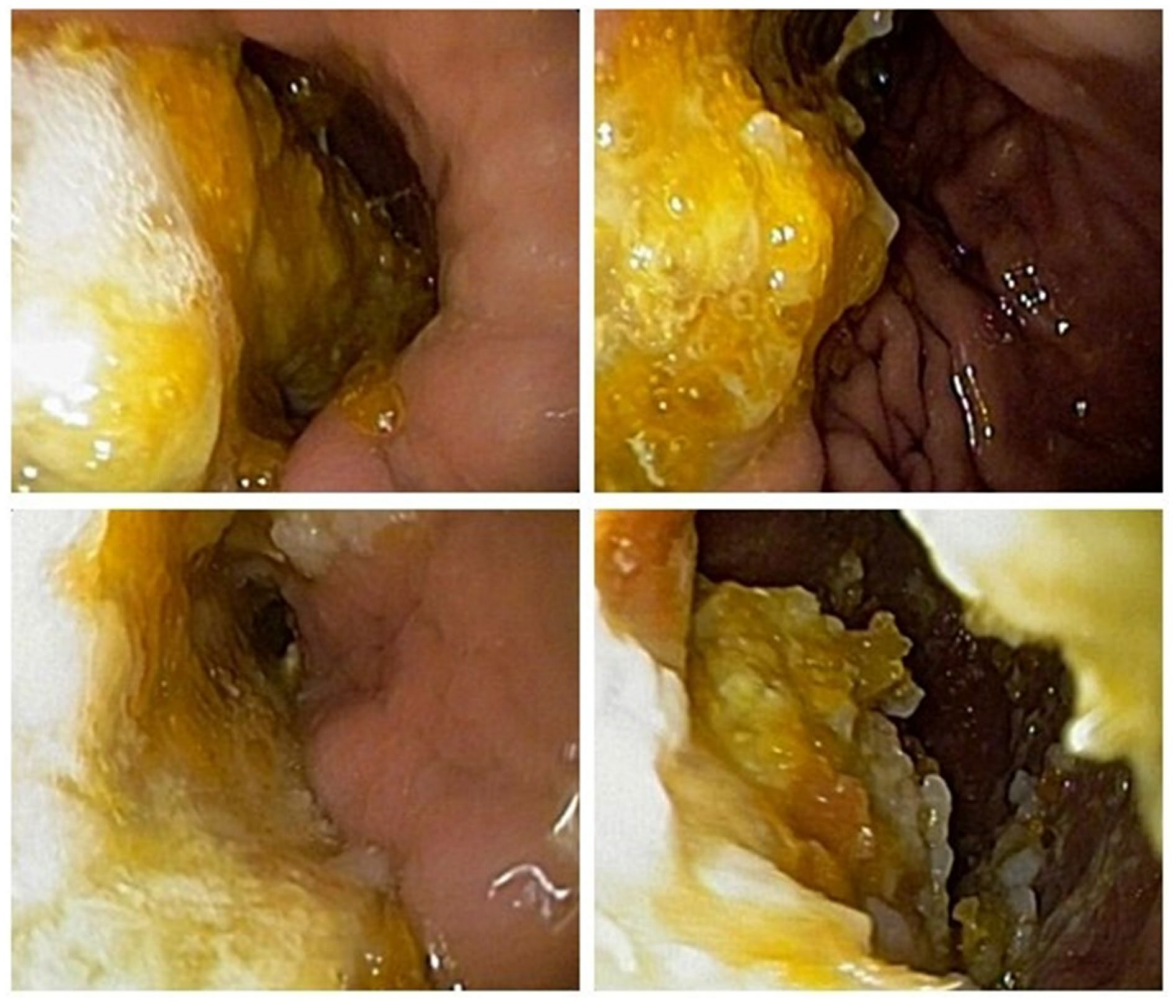
Gastroscopy presented plenty of food particles, indicating gastric retention.

Blood routine examination indicated decreased platelet count 91 × 10^9^/L. Antinuclear antibody was 1:100 (+) and anti-RO52 antibody was positive (+++). The following tests did not show any abnormalities: liver function, kidney function, fasting blood glucose, glycated hemoglobin, blood lipids, electrolytes, myocardial markers, high-sensitivity C-reactive protein, infectious disease tests, thyroid function and antibodies, tumor markers, serum creatine kinase, anti-streptolysin O, rheumatoid factor, lupus anticoagulant substance detection, anti-citrullinated peptide antibody measurement, complement tests, stool and urine routines. The anemia three items containing folic acid, vitamin 12 and serum ferritin were normal. Autoimmune antibodies, such as anti-SSA/SSB, anti-Sm, anti-Jo-1, anti dsDNA, anti-U1-RNP, anti-Scl-70, were negative. Dynamic ECG showed sinus rhythm with some atrial premature beats and short episodes of atrial tachycardia. Chest CT revealed some inflammatory lesions. Cardiac ultrasound, abdominal ultrasound, pelvic and urinary system ultrasound showed no significant abnormalities. Cranial MRI plain scan and vascular imaging showed no definite abnormalities. Electrophysiological tests including nerve conduction velocity, F wave and H reflex measurements, showed that the motor and sensory nerves of the limbs were normal. Salivary gland imaging showed no significant abnormalities in bilateral parotid glands and submandibular glands. In fact, this patient has not complained of dry mouth and eyes. The subsequent Saxon test and labial glands biopsy were refused by the patient and not scheduled. Gastroscopy presented plenty of food particles, indicating gastric retention (Figure 2). Whole-body PET-CT showed no definite signs of malignant lesions.

In consideration of obvious autonomic dysfunction, we have detected the serum gAChR-Ab, which was 0.3343 nmol/L (normal serum reference value: 0.05 nmol/L). Therefore, the diagnosis of AAG was suspected. The patient received intravenous infusion treatment of methylprednisolone 1,000 mg once daily for 3 days, followed by 500 mg/d for 3 days, and then 250 mg/d for 3 days. After that, the dosage was reduced to oral prednisone 60 mg/d. The patient’s syncope symptoms alleviated on the third day of medication. Remeasurement of blood pressure showed the supine blood pressure of 135/85 mmHg, immediate standing blood pressure of 125/78 mmHg, and standing after 3 min blood pressure of 82/57 mmHg. The patient had slight residual dizziness after activity, and was later discharged. After discharge, the dosage of oral prednisone was reduced by 5 mg weekly, and gradually decreased to a maintenance dose of 10 mg/d. During the medication period, the patient stopped worrying about the effect of syncope. In addition, bowel movements became more regular. However, a follow-up gastroscopy still indicated gastric retention after 6 months.

### Second stage

In March 2023, the patient visited the Department of Gastroenterology again due to “gastric retention.” The carbon 14 breath test was positive, indicating *Helicobacter pylori* (*H. pylori*) infection. Then, a tetralogy therapy for *H. pylori* was initiated, while corticosteroid treatment was stopped in the meantime. In early April 2023, the patient experienced repeated fainting accompanied by loss of consciousness and was admitted to our department. Upon admission, physical examination showed the supine blood pressure of 115/84 mmHg, and the immediate standing blood pressure of 45/30 mmHg. The serum gAChR-Ab level was 0.3824 nmol/L, suggesting a recurrence of AAG. The patient once again received methylprednisolone pulse therapy, and the dosage was as mentioned before. However, the patient fainted again the day after stopping intravenous corticosteroids, with the supine blood pressure of 90/65 mmHg, the immediate standing blood pressure of 68/45 mmHg and standing after 3 min blood pressure of 48/32 mmHg. For further immunotherapy, the patient underwent the double filtration plasmapheresis (DFPP) treatment on 27 April, 29 April, and 4 May 2023, respectively. After three times of DFPP, the patient was able to get out of bed and move around without experiencing fainting. Remeasurement of blood pressure showed the supine blood pressure of 120/75 mmHg, immediate standing blood pressure of 104/62 mmHg, and standing after 3 min blood pressure of 96/56 mmHg. After discharge, the patient was prescribed oral prednisone 80 mg/d plus mycophenolate mofetil 0.5 g bid. The dosage of oral prednisone was reduced by 5 mg weekly, and gradually decreased to a maintenance dose of 5 mg/d. No recurrence happened during follow-up for 2 months. The medication regimen was changed to prednisone 5 mg qod and mycophenolate mofetil 0.5 g/d for maintenance treatment until now. The patient can fully take care of herself in daily life, with no significant dizziness. The pupils were still dilated, but the pupillary reaction to light shown some improvement, especially the left side (Figures 1C,D). During follow-up gastroscopy, gastric retention was still observed, but there were no symptoms of nausea or vomiting. The patient was satisfied with the therapeutic effect and temporarily refused to retest serum gAChR-Ab level during follow-up.

## Discussion

Autoimmune autonomic ganglionopathy is relatively rare and there is no definitive epidemiological data available domestically or internationally. The median age at onset for AAG is 45–61 years old, and the ratio of male to female patients is about 1:2 (4, 5). A newly discovered syndrome described as “pure pan-dysautonomia” was first reported by Young et al. in 1969, and later classified along with other autonomic neuropathy as a part of the Guillain-Barré syndrome spectrum (6, 7). The detection of the pathogenic gAChR-Ab is helpful for identifying patients with various forms of autoimmune autonomic neuropathy and establishing AAG as an independent disease entity (8).

The gAChRs and muscle-type AChRs both belong to the nicotinic AChRs (nAChRs), which are ligand-gated cation channels are widely located in the central and peripheral nervous system (2). Muscle-type AChRs contribute to transmit motor neuron impulses through the neuromuscular junctions, and antibodies against the muscle AChR cause weakness in voluntary muscles in patients with myasthenia gravis (9). Different from muscle-type AChRs, the gAChRs mediate synaptic transmission in the peripheral autonomic ganglia. A previous study has confirmed that patients with higher antibody titers have wide spread autonomic failure (10). The gAChRs are composed of two α3 subunits and three other AChR subunits (typically β4), and pathogenic gAChR-Abs mainly combine with α3 subunits (2, 11). Some studies also paid attention to the detection of anti-β4 gAChR antibodies in AAG patients (12). Compared with gAChR-α3 antibodies, seropositive gAChR-β4 antibodies resulted in more frequent autonomic and extra-autonomic manifestations, including sensory disturbance, central nervous system involvement, endocrine disorders, autoimmune diseases, and tumors, reported in a diagnosed cohort involving 179 patients with AAG in Japan (13). In the present case, subunit-specific autoantibodies were verified to target the gAChR-α3 and the level was higher than the normal reference value.

The classic presentation of AAG is acute to subacute, usually with preceding events. However, some patients had chronic or insidious symptom onset with gradual progression (4). Patients typically present with extensive damage of sympathetic, parasympathetic and enteric systems, characterized by the most common orthostatic hypotension and prominent cholinergic failure, including sicca syndrome, abnormal pupillary light response, anhidrosis, upper and lower gastrointestinal dysfunction and neurogenic bladder (5). In the present report, the patient presented with chronic gastrointestinal dysfunction onset with alternating diarrhea and constipation, making it difficult to trace the antecedent events. The patient initially exhibited position-related dizziness, which was not specific. Her symptoms were not highly regarded until orthostatic syncope occurred. After admission and measuring blood pressure in different positions, severe orthostatic hypotension was discovered. Therefore, in general, orthostatic hypotension syncope is the direct reason for the patient’s visit and is the key diagnostic clue. After treatment, the patient’s syncope and standing blood pressure improved rapidly and daily living abilities were restored, which can serve as one of the early indicators for assessing the condition and treatment response.

Gastrointestinal dysfunction is another symptom seriously affecting patients daily living and working. In this case, the patient presented with alternating symptoms of diarrhea and constipation, and intermittent vomiting, which were often neglected symptoms related to AAG. Repeated visits to the Department of Gastroenterology and multiple gastrointestinal endoscopies yielded poor medication effects. It suggests that patients with unexplained gastrointestinal dysfunction should be evaluated for autonomic nervous system disorders. After treatment, the patient’s gastrointestinal symptoms significantly improved, but a follow-up gastroscopy indicated that gastric retention still existed, suggesting that gastrointestinal function requires a longer recovery time. Pupillary abnormalities are another common pathological change in antibody-positive AAG. According to research findings, premature pupil redilation was seen in patients with AAG, as the unique manifestation of pupillary constriction fatigue (14). Rigid pupils are one of the main signs in our patient, which do not yet affect vision. After treatment, the pupillary function shown some improvement, especially the left side. To investigate whether this patient had other autoimmune diseases, we conducted relevant examinations and found that antinuclear antibody and anti-RO52 antibody was positive, but there was no clear damage to target organs such as the kidneys. Whether these immunologic abnormalities would develop into other autoimmune diseases required long-term follow-up. Autonomic neuropathies can manifest as paraneoplastic syndromes or occur secondary to neoplasms (15). A previous study has reported that dual immune checkpoint inhibitor therapy in a patient with metastatic melanoma caused seronegative AAG (16). In order to exclude tumor-associated autonomic neuropathy, PET-CT and other examinations were carried out and did not reveal clear signs of tumors.

There are currently no diagnostic criteria of AAG. Clinical diagnosis is mainly based on the typical clinical manifestations after severe impairment of sympathetic and parasympathetic nervous systems, and some corresponding auxiliary examinations, while reasonably excluding other diseases for differential diagnosis. A previous study showed approximate 80% of AAG patients suffered orthostatic hypotension and orthostatic intolerance, and almost 75% of AAG patients presented with lower gastrointestinal dysmotility like constipation (13, 17). Additionally, objectively examined orthostatic hypotension and gastric retention provided diagnostic support. Many neurological and systemic diseases can display different forms of autonomic dysfunction, then gAChR antibody testing can serve as an important differential basis. In this case, the patient’s two gAChR-Ab measurements were 0.3343 nmol/L and 0.3824 nmol/L, both higher than the normal reference value. There are still some ways to test autonomic nerves system function when lacking of subjective symptoms of autonomic neuropathy. The head-up tilt test, the measurement of plasma catecholamine concentrations, the thermoregulatory sweat test, the conjunctival instillation test, and cardiac MIBG imaging are used to test sympathetic nervous system function, while tests for parasympathetic nervous system function include the Saxon test, the Schirmer test, and the conjunctival instillation test (17). By completing relevant examinations, we excluded common diseases that cause autonomic dysfunction, such as vitamin B12 deficiency, diabetes, connective tissue diseases, Guillain-Barré syndrome, and paraneoplastic diseases. These existing clinical data did not support the diagnosis of amyloidosis. Combined with severe autonomic dysfunction and positive gAChR antibodies, the diagnosis of AAG was established. Since the patient was admitted with serious symptoms and responded well to treatment, no further relevant examinations for other autonomic dysfunctions were conducted.

Autoimmune autonomic ganglionopathy AAG is a severe and potentially treatable disorder that responds well to immunotherapy, supporting the autoimmune pathogenesis of AAG, although evidence was limited to case reports and small cohorts (18). Immunomodulatory treatment, including plasma exchange, intravenous immunoglobulin and immunosuppressant agent, and a combined therapy can be usually effective in both seropositive and seronegative AAG (19). Some patients with refractory AAG who did not respond well to plasma exchange or intravenous immunoglobulin monotherapy, or relapsed might require prolonged immunotherapy such as mycophenolate mofetil or rituximab to sustain the clinical improvement (19). A study in Japan involving 31 patients with AAG showed that intravenous immunoglobulin, intravenous methylprednisolone and plasma exchange were generally regarded as the effective first-line therapy, and subsequent oral administration of prednisolone and/or immunosuppressants was positioned as second-line therapy (20). However, few patients receiving oral administration of immunosuppressants and curative effect was not clear. The patient reported in this article responded well after the first stage of methylprednisolone pulse therapy. Thereafter, a low-dose prednisone was administered to stabilize the clinical condition. However, AAG relapsed after stopping oral prednisone for 1 month, which might be regarded as a reference duration of hormone discontinuance. In the second stage, methylprednisolone pulse therapy provided little benefit. Fortunately, the symptoms were quickly alleviated with the addition of DFPP, one method of the plasma exchange. Subsequently, low-dose oral prednisone and mycophenolate mofetil were used for sustaining the clinical improvement. The experience in this case suggested plasma exchange could improve the “crisis” symptoms of AAG more quickly and sustainably to help transition to immune maintenance therapy.

This case report has several limitations. First, we have not retest serum gAChR-Ab levels after the first and second stage of treatment, which was hard to assess the correlation of antibody titers and severity of AAG. Second, the head-up tilt test, cardiac MIBG imaging and the plasma catecholamine levels have not yet been carried out in our hospital. Third, the possibility of sicca syndrome could not be completely excluded because the Saxon test and labial glands biopsy were not done. Despite these limitations, it has not affected the diagnosis and treatment of this present patient. There will be more emphasis on the comprehensive assessment of autonomic nervous function in the future.

Autoimmune autonomic ganglionopathy is a disorder defined by antibodies to the gAChR, characterized by symptoms of autonomic failure, including orthostatic hypotension, sicca syndrome, abnormal pupillary light response, anhidrosis, upper and lower gastrointestinal dysfunction and neurogenic bladder. The initial signs of AAG are easily ignored, therefore, the disease may first be diagnosed in related departments such as gastroenterology, cardiology, neurology, and ophthalmology. Attention should be paid to the collection of relevant medical history and assessment of autonomic dysfunction. If patients present with unexplained autonomic dysfunction, the possibility of AAG should be considered. About half of the patients may test positive antibodies to the gAChR, but a negative result does not rule out the disease. AAG responds well to the conventional immunotherapy, and some patients may require a long-term immunotherapy. The reduction and withdrawal of immunosuppressants should be slow and cautious.

## Data Availability

The original contributions presented in the study are included in the article/supplementary material, further inquiries can be directed to the corresponding authors.
